# The Impact of Cytokines Expression in Peri-implant Crevicular Fluid Associated with Keratinized Tissue Augmentation via Xenogeneic Collagen Matrix: A Pilot Study

**DOI:** 10.1055/s-0045-1813033

**Published:** 2026-01-19

**Authors:** Xin-Rui Zhu, Ying-Jie Lin, Chang Chen, Rui-Yong Wang, Yi Liu

**Affiliations:** 1 Department of Periodontics, School of Stomatology, Capital Medical University, Beijing, People's Republic of China; 2 Department of Stomatology, Beijing Haidian Hospital, Beijing, People's Republic of China; 3 Department of Oral Healthcare, Chinese Stomatological Association, Beijing, People's Republic of China

**Keywords:** peri-implant crevicular fluid, cytokine, dental implant, keratinized tissue augmentation, xenogeneic collagen matrix

## Abstract

**Objective:**

The xenogeneic collagen matrix (XCM) is widely used for keratinized mucosa augmentation around natural teeth and dental implants due to its consistent efficacy and the advantage of avoiding a second surgical site. However, the shrinkage rate of XCM after surgery exceeds 50%, which is not conducive to accurate preoperative design. This study aimed to investigate factors influencing XCM shrinkage.

**Materials and Methods:**

Fifteen participants with buccal keratinized tissue width (KTW) <2 mm around mandibular single implants underwent XCM-based KTW augmentation. Clinical parameters were recorded, and peri-implant crevicular fluid (PICF) was collected preoperatively and at 3-month follow-ups. Twenty cytokines in PICF were assessed using a commercial kit, with Spearman tests evaluating correlations between cytokines, clinical parameters, and shrinkage predictors.

**Results:**

XCM significantly increased KTW from 1.39 ± 0.26 mm to 4.13 ± 1.19 mm at 3 months ( *p* < 0.001), with a shrinkage rate of 60.12 ± 12%. The PICF showed significant decreases in C-reactive protein (CRP), interleukin-6 (IL-6), calprotectin (CLP), and keratin type II cytoskeletal 1 (K2C1) ( *p* < 0.05) and a significant increase in osteoprotegerin (OPG) at 3 months ( *p* < 0.05). Preoperative keratinized tissue thickness (KTT) and cathepsin K (CTSK), along with K2C1 at 3 months, correlated with XCM shrinkage.

**Conclusion:**

KTW augmentation alters cytokine expression. Thin preoperative KTT and high CTSK in PICF may predict high postoperative XCM shrinkage.

**Clinical Relevance:**

Gingival phenotype significantly impacts XCM shrinkage after augmentation. PICF cytokine expression could serve as a predictive biomarker.

**Trial Registration:**

The Chinese Clinical Trial Registry, no. ChiCTR2500100933 (last updated on 17/04/2025).

## Introduction

Keratinized tissue width (KTW) plays a pivotal role in ensuring the long-term stability and retention of both natural teeth and dental implants. 1 2 3 4 5 6 Prior research has demonstrated that a KTW of less than 2 mm is prone to plaque accumulation, soft tissue inflammation, and recession. These conditions can subsequently lead to localized bleeding, pain, diminished patient satisfaction, and ultimately, implant failure. 7 The combination of an apically positioned flap (APF) with a free gingival graft (FGG) is widely regarded as the gold standard for augmenting KTW. Nonetheless, the procurement of FGG necessitates a second surgical site, which frequently gives rise to complications such as heightened postoperative pain, excessive bleeding, and localized numbness. These complications, in turn, restrict the widespread application of this technique.

In recent years, researchers have conducted extensive investigations into various substitutes for FGG, aiming to diminish the complexity of soft tissue augmentation procedures and enhance patient satisfaction. Notably, numerous studies have demonstrated that the xenogeneic collagen matrix (XCM) can substantially augment KTW. However, XCM is associated with a high shrinkage rate, which complicates surgeons' ability to predict the initial graft volume required; its extensive variability undermines the precision of keratinized tissue augmentation. 8 Identifying the factors that influence the contraction rates of XCM could offer potential solutions to these challenges.

Current research on keratinized gingival shrinkage focuses primarily on autologous tissue. Some studies have reported that different gingival biotypes impact the postoperative shrinkage rate of FGG transplantation; however, their effect on XCM has not been reported. 9 Previous studies have shown that patients with a thin gingival biotype are more likely to experience gingival recession than those with a thick biotype, which may be related to thinner alveolar bone, fewer blood vessels, less fibrous tissue, and poorer resistance to minor chronic irritation or trauma. 10 11 Wound healing and tissue remodeling in the periodontium involve numerous cell-to-cell and cell-to-extracellular matrix interactions mediated by chemokines, cytokines, enzymes, and growth factors. 12 13 Peri-implant crevicular fluid (PICF) is the serum exudate of healthy individuals or inflammatory exudate composed of peri-implant tissue cells, serum, and oral bacteria, which contains cytokines. 14 Unlike serum and saliva, which lack site-specificity, PICF is released in close proximity to the peri-implant tissues and therefore allows for a more objective assessment of mucogingival surgery. 15 However, the role of cytokines in affecting the shrinkage rate of XCM remains unclear. This study analyzed 20 cytokines, including 11 inflammatory factors, 5 mechanical factors, 2 osteoclast-related factors, and 2 epithelial cell stability-related factors. Previous studies have shown that these cytokines may be related to the outcomes of mucogingival surgery. 12 16

This study aimed to preliminarily evaluate the factors related to XCM shrinkage rate through changes in clinical indicators and cytokine profiles.

## Materials and Methods

### Ethical Considerations

The present study was designed as a pilot study to explore factors affecting XCM shrinkage. It was approved by the Clinical Research Ethics Committee of Beijing Haidian Hospital (Grant no. BHHMEC-XM-2022–21) and conducted in accordance with the 2013 revised version of the Declaration of Helsinki. All participants provided written informed consent before enrollment.

### Inclusion Criteria

The inclusion criteria were as follows: (1) age between 18 and 60 years; (2) nonpregnant or breastfeeding; (3) buccal KTW < 2 mm around a single dental implant in the mandibular region; (4) self-reported non-smoking status; (5) good oral hygiene with bleeding on probing (BOP) < 20%; (6) extraoral bonding for implant restoration.

### Exclusion Criteria

The exclusion criteria included (1) untreated periodontal disease; (2) prior surgical history at the surgical site; (3) allergy to collagen, cephalosporins, loxoprofen, or chlorhexidine mouthwash; (4) untreated dental caries adjacent to the surgical site; (5) participation in another clinical study within the past six months; (6) uncontrolled systemic diseases; (7) less than 3 months since implant restoration 17 ; (8) long-term use of nonsteroidal anti-inflammatory drugs or analgesics; (9) peri-implant mucositis or peri-implantitis in the surgical area; (10) less than 1 mm of cortical bone on the buccal side of the implant.

### Study Design and Patient Groups

This study employed a consecutive enrollment protocol and included all 243 patients who underwent implant restoration at our institution between January 2022 and December 2023. All patients received titanium screw-type bone-level implants from Straumann. Based on the inclusion and exclusion criteria, a total of 48 patients were screened and met the criteria. Ultimately, 15 patients agreed to participate and signed informed consent forms for subsequent research. The study was conducted in accordance with the CONSORT (CONsolidated Standards of Reporting Trials) guidelines.

### Surgical Procedure and Follow-up

**Fig. 1:**
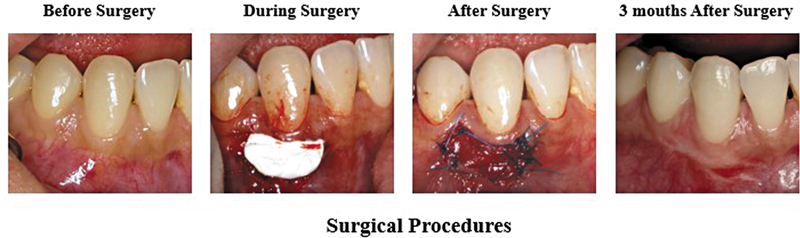
Clinical photographs for surgical procedures of increasing keratinized tissue width with XCM.

All patients underwent keratinized tissue augmentation with an XCM (Mucograft; Geistlich Pharma AG, Wolhusen, Switzerland) performed by the same surgeon (Dr. Zhu). One week before surgery, the patient received a full mouth cleaning and oral hygiene education. The surgical procedure was as follows: local infiltration anesthesia was administered at the surgical site. A horizontal incision was made 1 mm above the mucogingival junction using a 15C blade, and a partial-thickness flap was elevated. The partial thickness flap was secured at the recipient site using 5–0 absorbable sutures, positioned 9 mm from the gingival margin. The XCM was trimmed to match the size of the recipient site and fixed tension-free using 5–0 non-absorbable sutures, employing a combination of interrupted and horizontal mattress sutures ( Fig. 1 ). All patients were advised to apply ice packs to the surgical site within the first 6 hours following surgery. They were prescribed 250 mg of cefuroxime axetil every 12 hours for 6 days, along with 400 mg of loxoprofen for pain relief as needed. During the initial 2-week period, patients were instructed to refrain from brushing the surgical site and to avoid hard foods to prevent mechanical injury. Instead, they were advised to consume a soft-food diet, brush the nonsurgical areas twice daily with a soft-bristled toothbrush, and rinse with 0.12% chlorhexidine mouthwash three times daily for one minute each time. Sutures were routinely removed after 14 days.

### Clinical Evaluation

The collection and analysis of clinical and biological sample data were performed at pre-surgery (T _0_ ), after-surgery (T _1_ ), and 3 months after surgery (T _2_ ) (Dr. Lin). The following parameters were recorded: (1) KTW—measured as the vertical distance from the midpoint of the buccal gingival margin of the implant restoration to the mucogingival junction using a periodontal probe. (2) Keratinized tissue thickness (KTT)—assessed by inserting an endodontic file with a rubber stopper into the midpoint of the vertical distance from the midpoint of the buccal gingival margin of the implant restoration, which at the surgical site, to the mucogingival junction until the tip contacted the bone surface. After positioning the rubber stopper against the gingiva, the endodontic file was removed, and the distance from the tip to the rubber stopper was measured using a digital caliper with an accuracy of 0.1 mm. 18 (3) KTW shrinkage rate: calculated as [KTWT _1_ − KTWT _2_ ] / KTWT _1_ × 100. 19

### Peri-implant Crevicular Fluid Samples

Following the removal of plaque and subsequent drying of the target sites, six sterile Whatman 3 MM filter papers were gently inserted into the peri-implant crevicular areas of the selected implants (mesiobuccal, midbuccal, distobuccal, mesiopalatal/lingual, midpalatal/lingual, and distopalatal/lingual) for a duration of 30 seconds. This standardized collection period, based on previous research and laboratory assessments, ensured compliance with the manufacturer's instructions for the cytokine detection process. 14 The samples were then transferred individually to sterile 1.5 mL tubes and stored at −80°C until further use. 13

### Cytokine Detection

The PICF collected on the filter papers was diluted with 500 µL of sample diluent provided with the Human Cytokine Antibody Array Kit (QAH-PDD-1, RayBiotech, United States). This kit is microbead-based and has a detection threshold of 150 pg/mL. The protein concentrations of all samples were measured by the BCA method. To detect specific cytokine concentrations, all samples were further diluted to a final concentration of 300 μg/mL. Then, a total of 60 µL from each sample was added to the array. Twenty cytokines, including C-reactive protein (CRP), tumor necrosis factor-α (TNF-α), TNF-β, interleukin (IL)-1, IL-2, IL-6, IL-10, IL-17, matrix metalloproteinase (MMP)-1, MMP-8, MMP-9, MMP-13, macrophage inflammatory protein-2 (MIP-2), cathepsin K (CTSK), osteoprotegerin (OPG), RANK ligand (RANKL), calprotectin (CLP), keratin type II cytoskeletal 1 (K2C1), extracellular calcium-sensing receptor (CASR), and S100A9 were detected according to the manufacturer's instructions.

Briefly, the arrays were incubated with PICF samples for 2 hours at room temperature (RT) after blocking. The arrays were then washed with PBS three times and incubated with biotin-conjugated antibodies for 2 hours at RT. Subsequently, after additional washing with PBS, the arrays were incubated with Cy3-equivalent dye-conjugated streptavidin in the dark for 1 hour at RT. Finally, the arrays were visualized using a laser scanner, and the data were analyzed with Axon GenePix software. 20 Cytokine testing was performed with assistance from a third-party company.

### Statistical Analysis

The normality of numerical data was assessed using the Kolmogorov–Smirnov test and the Shapiro–Wilk test. Repeated measures ANOVA was used for data that met the criteria for a normal distribution. For non-normally distributed data, Friedman's test was used for comparisons. Spearman's correlation coefficient was used to evaluate the association between cytokine levels and the shrinkage rate of KTW. A *p* -value less than or equal to 0.05, determined using SPSS 23.0 (IBM, United States), was considered statistically significant.

## Results

### Study Sample

**Fig. 2:**
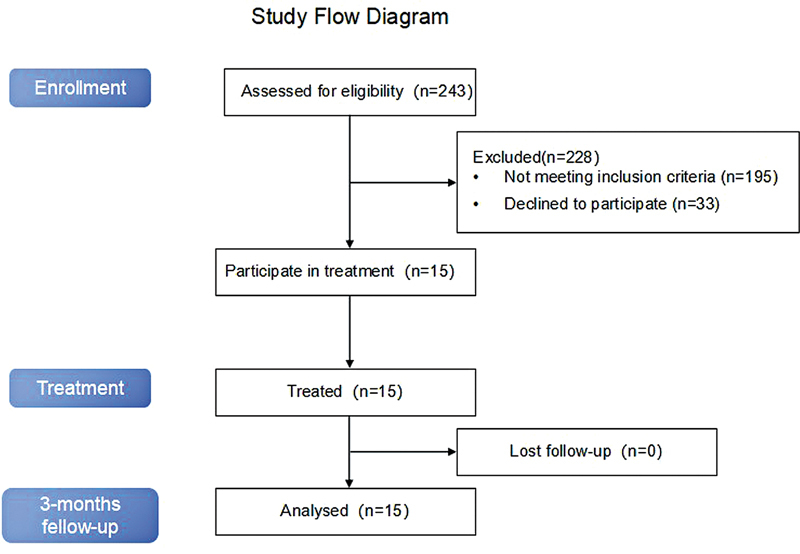
Workflow of the study.

After the enrollment stage, 15 patients were included in this study ( Fig. 2 ). The baseline characteristics of the study participants are presented in Table 1 .

**Table 1:** Baseline characteristics of the study participants

Variable	Patients ( *n* = 15)
Age (years), mean ± SD	48.1 ± 6.7
Gender (M/F), no.	8/7
BMI (kg/m ^2^ ), mean ± SD	22.5 ± 2.4
Probing depth (mm), mean ± SD	3.1 ± 1.0
Bleeding on probing (%), mean ± SD	4.8 ± 3.1
Keratinized tissue width (mm), mean ± SD	1.4 ± 0.3
Keratinized tissue thickness (mm), mean ± SD	1.1 ± 0.4

### Primary Outcome

The mean rate of KTW was 10.39 ± 0.26 (SD) mm at T _1_ and decreased to 4.13 ± 1.19 (SD) mm at T _2_ . Those changes in KTW were significant compared with T _0_ ( *p* < 0.001). The shrinkage rate of XCM was 60.12 ± 12 (SD)%. The changes in KTT ( *p* = 0.173), BOP ( *p* = 0.774), and probing depth (PPD, *p* = 0.934) did not show significant differences.

Protein expression levels in PICF at T _0_ and T _2_ are presented in Table 2 . Compared with T _0_ , the expression of CRP ( *p* < 0.05), IL-6 ( *p* < 0.05), CLP ( *p* < 0.05), and K2C1 ( *p* < 0.05) was significantly decreased at T _2_ . Additionally, the expression of OPG ( *p* < 0.05) was significantly increased.

**Table 2:** Cytokines expression in PICF changes at baseline (T _0_ ) and after 3-month follow-up session (T _2_ )

Cytokine (pg)	Baseline (T0, *n* = 15)				3 mo after surgery (T2, *n* = 15)				*p*
	Mean	SD	Median	IQR	Mean	SD	median	IQR	
CRP	239,038.72	27,717.41	229,706.88	(221,119.03–262,572.40)	227,841.43	28,932.75	222882.86	(204543.91-249408.42)	0.041 a
TNF-α	127,341.03	22,830.42	124,133.63	(107,429.34–141,927.72)	133,538.21	22,687.51	126929.75	(118409.71–146869.59)	0.066
TNF-β	18,041.42	3,804.16	16,686.72	(14,242.19–22,029.06)	18,518.17	4,260.13	18995.29	(14431.68–22151.16)	0.615
IL-1	112,193.81	16,809.41	117,756.74	(95,560.41–124,996.45)	101,756.92	13,615.02	97852.92	(94119.67–115482.71	0.068
IL-2	91,212.93	7,063.74	92,585.04	(86,259.68–96,377.84)	86,385.50	8,419.65	83362.42	(79784.72–96879.26)	0.112
IL-6	28,472.95	3,877.81	28,028.49	(26,545.93–30,853.08)	26,163.00	5,265.02	28246.56	(20324.31–30791.7)	0.043 a
IL-10	2,063.55	625.86	2,255.88	(1,398.12–2,665.54)	2,040.77	626.40	2043.19	(1546.18–2574.96)	0.786
IL-17	2,235.77	688.69	2,410.11	(1,423.7–2,795.93)	2,407.54	571.56	2648.15	(2007.49–2855.91)	0.132
MMP-1	147,512.40	19,286.24	153,225.88	(136,109.19–164,700.57)	143,739.08	18,794.15	143610.70	(123962.96–159200.24)	0.246
MMP-8	180,958.22	19,823.91	183,924.33	(163,862.78–195,469.33)	180,905.00	25,726.59	184002.62	(160240.66–198070.02)	0.989
MMP-9	181,411.90	42,882.37	191,661.70	(141,121.31–223,232.63)	180,023.11	40,147.74	190057.75	(146784.48–199382.08)	0.767
MMP-13	82,332.55	2,274.05	78,448.86	(57,227.72–97,997.31)	82,016.61	2,003.93	84035.53	(68491.09–87931.03	0.949
MIP-2	3,841.22	828.84	3,765.08	(3,057.49–4,510.63)	3,863.26	712.05	4014.80	(3080.92–4224.05)	0.839
CTSK	4,158.58	1,170.26	4,088.43	(3,095.21–5,201.27)	4,385.24	984.33	4251.82	(3783.04–5452.2)	0.170
OPG	17,602.29	2,416.60	17,872.26	(1,496.03–1,952.34)	19,511.50	3,455.41	19566.75	(17255.96–22066.87)	0.048 a
RANKL	20,158.83	5,535.63	21,042.06	(15,622.24–25,625.89)	19,664.75	4,308.18	20550.02	(15818.40–22981.01)	0.638
CLP	455.18	70.20	481.53	(392.26–519.82)	393.49	74.56	351.07	(337.66–456.8)	0.005 a
K2C1	409.97	90.01	405.07	(318.62–488.04)	344.44	104.43	309.45	(254.52–443.74)	0.024 a
CASR	2,046.60	352.67	2,125.08	(1,627.61–2,369.27)	2,140.97	400.64	2117.15	(1803.36–2548.28)	0.421
S100A9	116.82	48.98	106.41	(75.85–165.24)	141.94	48.85	141.77	(103.22–184.16)	0.093

Note: Values are represented as mean ± SD and median with interquartile range (IQR).

a *p* < 0.05.

### Clinical and Cytokine Variables Stratified for Shrinkage Rate

The correlation analysis regarding the shrinkage rate is presented in Table 3 . Clinical variables indicated a significant correlation between the shrinkage rate and KTT at T _0_ (rs = −0.797; *p* < 0.001) and at T _2_ (rs = − 0.86; *p* < 0.001). Additionally, the amounts of KTW, PPD, and BOP showed minimal correlation with the shrinkage rate.

**Table 3:** Correlation analysis at baseline (T _0_ ) and after 3 months follow-up session (T _2_ ) among the shrinkage rate and clinical or cytokine variables

Variable	Baseline (T _0_ , *n* = 15)		3 months after surgery (T _2_ , *n* = 15)	
	rs coeff.	*p-* Value	rs coeff.	*p-* Value
KTW	0.288	0.298	−0.989	<0.001 a
KTT	−0.797	<0.001 a	−0.86	<0.001 a
PPD	−0.098	0.727	−0.193	0.491
BOP	0.129	0.648	0.237	0.395
CRP	−0.171	0.541	−0.154	0.585
TNF-α	−0.211	0.451	−0.3	0.277
TNF-β	0.182	0.516	−0.007	0.98
IL-1	0.229	0.413	−0.361	0.187
IL-2	0.232	0.405	−0.357	0.191
IL-6	0.146	0.603	0.175	0.533
IL-10	−0.304	0.271	−0.507	0.054
IL-17	0.196	0.483	0.304	0.271
MMP-1	−0.057	0.84	0.082	0.771
MMP-8	−0.018	0.95	−0.368	0.177
MMP-9	0.111	0.694	0.096	0.732
MMP-13	−0.232	0.405	0.104	0.713
MIP-2	−0.171	0.541	0.004	0.99
CTSK	0.55	0.034 a	0.432	0.108
OPG	0.45	0.092	0.261	0.348
RANKL	0.121	0.666	0.264	0.341
CLP	−0.439	0.101	−0.436	0.104
K2C1	0.093	0.742	0.532	0.041 a
CASR	−0.336	0.221	−0.496	0.06
S100A9	−0.125	0.657	−0.189	0.499

a *p* < 0.05.

The correlation analysis between the shrinkage rate and cytokines in PICF revealed a significant correlation with CTSK at T _0_ (rs = 0.55; *p* < 0.05) and K2C1 at T _2_ (rs = 0.532; *p* < 0.05).

## Discussion

Several studies have demonstrated that maintaining more than 2 mm of keratinized tissue around implants is crucial for preserving soft tissue health and reducing the incidence of inflammatory diseases. 21 22 In this study, the peri-implant KTW was measured, yielding a result of 4.13 ± 1.19 mm three months after surgery, indicating that the application of XCM can effectively increase KTW.

Reports on the shrinkage rate of XCM have shown that it can reach up to 70%. To ensure that KTW increased by at least 2 mm and to limit intra-group heterogeneity in this study, we standardized the XCM width to 9 mm. The observed shrinkage rate was

60.12 ± 12%, which is consistent with the results of most previous studies. 23 24 25 Additionally, we evaluated several preoperative clinical examination results and analyzed their correlation with postoperative XCM shrinkage. The results indicated that gingival thickness was significantly negatively correlated with gingival recession, suggesting that the gingival phenotype may play an important role in XCM shrinkage. When using XCM for subsequent KTW procedures, it is important to consider differences in gingival phenotype when determining the width of the attached peri-implant flap (APF). Furthermore, XCM had minimal effect on KTT, which aligns with previous reports. 26 These results further emphasize the importance of preoperative KTT.

In addition to clinical variables, we also measured cytokines in PICF before and three months after surgery. Previous studies have shown that the shrinkage rate of keratinized gingiva after XCM augmentation is primarily concentrated within the first three months. 24 Therefore, we chose to assess cytokines at this time point, when the shrinkage rate is at its highest, to establish a relationship with the shrinkage rate. The 20 factors included in this study are associated with soft tissue healing and can be detected three months after surgery. 12 16 The cytokines of PICF play an important role in providing primary defense against microorganisms and their products. 27 Furthermore, PICF demonstrates high sensitivity in reflecting alterations in periodontal status following oral therapeutic interventions. 28

Considering that cytokines are involved in an overlapping manner during healing, a wide spectrum of biomarkers should be analyzed for deeper insight into the ongoing wound healing process. The cytokine functions detected by the kit primarily include inflammatory cytokines, which can be divided into pro-inflammatory and anti-inflammatory categories. The expression balance between these cytokines reflects the inflammatory state around the implant. 29 Based on the results, the expressions of CRP and IL-6 decreased three months after surgery. Since both CRP and IL-6 are pro-inflammatory cytokines, these findings suggest that KTW augmentation may enhance the anti-inflammatory capacity of peri-implant soft tissue. Additionally, we observed that OPG increased and cathelicidin (CLP) decreased after surgery, indicating that KTW augmentation might also enhance bone protection and reduce bone resorption, thereby improving resistance to peri-implant diseases. 30 Moreover, factors reflecting epithelial cell stability were also included in the kit. Previous research has utilized K2C1 to reflect epithelial cell breakdown. 31 We noted a decrease in the expression of K2C1 at 3 months postsurgery, suggesting that KTW augmentation may contribute to the integrity and functional capacity of the keratinocyte barrier. 32

We also performed correlation analyses of clinical and cytokine variables related to the shrinkage rate. The results indicated that preoperative KTT significantly affected the shrinkage rate of XCM after surgery. Previous research has shown that individuals with a thin gingival phenotype typically exhibit thinner alveolar bone, fewer blood vessels, and reduced fibrous tissue, which are associated with a higher risk of periodontitis, peri-implantitis, gingival recession, and marginal bone loss (MBL) after implant insertion. 11 33 34 Furthermore, a thin gingival phenotype could influence the outcomes of dental treatments, such as non-surgical mechanical treatment for peri-implantitis. 30 In this study, we found that the gingival phenotype can impact the shrinkage rate of XCM following KTW augmentation, thereby affecting the treatment outcome. Additionally, the expression of CTSK before surgery may serve as a predictor of XCM shrinkage. Since CTSK plays a significant role in collagen fiber degradation within periodontal connective tissues, our results suggest that higher collagen fiber degradation activity may lead to increased XCM shrinkage. 35 This finding is further supported by the correlation between postoperative K2C1 expression and the XCM shrinkage rate. Considering that patients with thin gingiva have poor epithelial stability, the statistical differences observed in the aforementioned cytokines can also reflect the impact of KTT on the XCM shrinkage rate in clinical practice.

Several variables may influence the composition of PICF. First, analgesics and antibiotics can alter various aspects of inflammation and early healing after surgery in a dose- and type-dependent manner. 36 Consequently, this study required participants to use antibiotics and analgesics uniformly. Second, the impact of smoking on the proteome is complex. Blanco et al. 37 demonstrated that cytokine expressions differed between smokers and healthy individuals, characterized by increased expression of keratinization-related proteins. However, these changes in cytokines were not significantly different among healthy individuals, smokers, and ex-smokers. Therefore, we excluded smokers from this study. Third, the method of cementation may affect the composition of cytokines. Kıran et al 28 found higher expressions of OPG and IL-17 around crowns cemented extraorally compared with those cemented intraorally, indicating better osseointegration. Thus, all patients enrolled in this study underwent extraoral cementation. Lastly, all suture materials elicit varying degrees of inflammatory reactions in tissues and trigger the accumulation of neutrophils, which act as sources of cytokines and MMPs. 38 We used the same sutures for all participants.

However, the current study has several limitations. First, this is only a pilot study focused on the shrinkage rate of XCM during KTW augmentation. By correlating the clinical parameters of the surgical area before and after XCM augmentation of keratinized gingiva with changes in cytokine expression in PICF, factors affecting the shrinkage rate of XCM can be predicted. However, due to the small sample size included in this study, this analysis has insufficient statistical power. Subsequent clinical trials need to be designed based on the differential factors identified in this study to obtain definitive conclusions. Second, this is a short-term follow-up study, as most XCM shrinkage occurs within the first three months after surgery. The role of newly formed keratinized tissue in ensuring long-term implant stability remains to be fully elucidated. Third, Gurlek et al 39 compared the biomarkers in gingival crevicular fluid (GCF) and PICF, finding that IL-1β levels were significantly higher in PICF samples from healthy implants than in GCF samples from healthy teeth. This discrepancy suggests that the results of this study may only applicable to XCM-assisted KTW around implants.

## Conclusion

XCM-enlarged peri-implant keratinized gingiva exhibited significant shrinkage three months after surgery. A preoperative thin gingival phenotype and elevated CTSK expression levels in PICF may serve as potential predictors of XCM shrinkage.
